# Synthesis and electrochemical studies of NaCoPO_4_ as an efficient cathode material using natural deep eutectic solvents for aqueous rechargeable sodium-ion batteries

**DOI:** 10.3389/fchem.2024.1440639

**Published:** 2024-09-20

**Authors:** C. V. V. Eswara Rao, Sannapaneni Janardan, H. Manjunatha, K. Venkata Ratnam, Sandeesh Kumar, K. Chandrababu Naidu, Shivendu Ranjan

**Affiliations:** ^1^ Department of Chemistry, GITAM School of Science, GITAM University, Bengaluru, India; ^2^ Department of Physics, GITAM School of Science, GITAM University, Bengaluru, India; ^3^ School of Nanoscience and Technology, Indian Institute of Technology, Kharagpur, India

**Keywords:** deep eutectic solvent, rechargeable batteries, NaCoPO_4_, cathode, electrochemical studies

## Abstract

In this work, sodium cobalt phosphate (NaCoPO_4_) was successfully prepared by a cost-effective ionothermal method using a deep eutectic solvent (DES) for the first time. The synthesized NaCoPO_4_ was used to fabricate a cathode material for aqueous rechargeable sodium-ion batteries. The surface morphology of the prepared materials and its compositional analysis were done by using field emission scanning electron microscopy (FESEM) and energy-dispersive X-ray (EDX) analysis, respectively. The X-ray diffraction (XRD), SEM, and EDX studies revealed that the material has orthorhombic-shaped particle morphology with uniform distribution and is in nanoscale (approximately 50 nm). The nature of the cation inserted (Na^+^ ion insertion) was confirmed by recording CV profiles at different concentrations of the Na_2_SO_4_ electrolyte. The reversibility of the electrode redox reaction was studied by varying the scan rate in CV studies, and it was found that the electrode exhibits a reversible behavior with a resistive behavior. In GCPL studies, the cell TiO_2_/2MNa_2_SO_4_/NaCoPO_4_ showed significant reversibility with a prominent discharge capacity of 85 mAh g^−1^ at 0.1°C and 88% of capacity retention after 100 cycles. Thus, the prepared materials could be used as an effective futuristic alternative battery material for rechargeable batteries.

## Graphical Abstract

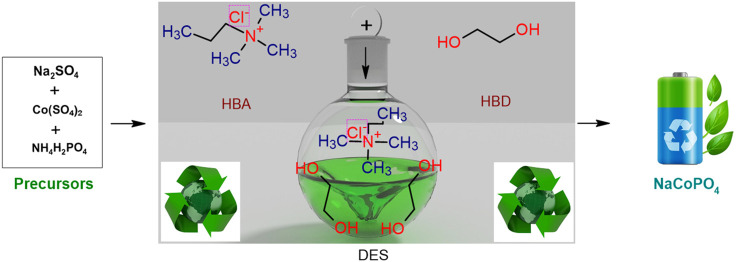

## 1 Introduction

Green technology-based energy storage systems have gathered significant attention in the world’s current energy scenario (Masias et al., 2021; Jin et al., 2021; Min et al., 2021; Bi et al., 2020). Lithium-ion batteries (LIBs) have been more commercialized and utilized in various applications, including electronic devices and electric vehicles (Chen et al., 2021; Chang et al., 2022). However, the low availability and high cost of lithium-based energy storage materials motivate scientists to search for new alternative energy sources. LIBs are not an excellent choice for the systems to store more energy in the case of large-scale applications like stationary energy storage devices (Lyu et al., 2020; Gao et al., 2018); however, sodium-ion-based batteries (SIBs) are the best alternatives and occupy a significant position due to their storage capacity (next to lithium), wide availability, low cost, and comparable electrochemical properties to lithium (Chen et al., 2022; Huang et al., 2022; Xia and Dahn, 2012). In lithium-ion batteries, cathode materials themselves occupy 25% of the total cost of the battery, where one can reduce 20%–30% of the price of the battery by replacing it with Na materials in lithium counterparts.

It is well known that portable, eco-friendly, and cost-effective energy storage materials are essential in the current commercial sector to meet the market demand in the automobile and industrial sectors. The prime advantage of Na-ion batteries comes from the natural abundance and the lower cost of Na over Li in the earth’s crust, i.e., 23,600 ppm–20 ppm. The cost of purification from the respective ores is significantly less in the case of Na compared to Li metal. LiCoPO_4_ and NaFePO_4_ synthesized through microwave-assisted synthesis were effectively used as electrode material and supercapacitors (Kreder et al., 2015; Zhu et al., 2013; Fang et al., 2015; Boddula et al., 2018; Bolagam et al., 2018; Fang et al., 2018; Fang et al., 2017; Jahne et al., 2013; Li et al., 2022; Lu et al., 2015; Qiu et al., 2021). It is effortlessly combined with abundantly available transition metal oxides and forms separate cathode materials such as NaCoO_2_ and NaFePO_4_. However, limited charge/discharge capacities (<10 mA h g^−1^) were observed for the NaCoPO_4_ (NCP) phase obtained through solid-state synthesis, and it is very low compared with its theoretical capacity, which is approximately 152 mAh g^−1^ (Ong et al., 2011; Gutierrez et al., 2017; Shiprath et al., 2020; Chiring et al., 2021; Shen et al., 2020; Song et al., 2013).

Ionic liquids (ILs) are significant in research areas like electrochemistry, gas separation, catalysis, lubricants, and biomass dissolution due to their unique properties, which include negligible vapor pressure, non-flammability, wide liquid range, high acid gas solubility, and thermal stability. All these properties have been exploited for different high-potential applications. One of the challenges with the use of ionic liquids is high prices. It is the main obstacle to their use. On the other hand, deep eutectic solvents (DESs) are an analog of ILs with various similar characteristic features. DESs are formed with a hydrogen-bond donor (HBD) and a hydrogen-bond acceptor (HBA) at a certain mole ratio. There is no need for further purification, and they are cheaper than traditional ILs. DESs can be used as efficient, simple, safe, and low-cost solvents and also possess physicochemical properties like phase behavior, density, viscosity, ionic conductivity, surface tension, and polarity. Recently, areas such as organic reactions, electrochemical processes, nanoscience, and pharmaceuticals have focused on DESs as novel green solvents (Wang et al., 2020; Achkar et al., 2021).

In this work, we used choline chloride and ethylene glycol-based DES at a ratio of 1:2 (Shiprath et al., 2021). It is a type-III DES solution. These type-III eutectics consist of quaternary ammonium salts as hydrogen-bond acceptors and ethylene glycol as the hydrogen-bond donor. This class consists of metal-free deep eutectic solvents (Achkar and Gerges Sophie, 2021). Thus, a choline chloride and ethylene glycol DES mixture was successfully used for the synthesis of NaCoPO_4_ nanomaterial, used as a cathode material for aqueous rechargeable sodium-ion batteries. To the best of our knowledge, this has not been reported so far.

## 2 Experimental procedure

### 2.1 Materials

All the chemicals, sodium sulfate (Na_2_SO_4_), cobalt sulfate (Co(SO_4_)_2_), ammonium dihydrogen phosphate (NH_4_H_2_PO_4_), choline chloride, and ethylene glycol were purchased from Sigma-Aldrich and used as such without any further purification.

### 2.2 Synthesis of deep eutectic solvents

**SCHEME 1:**
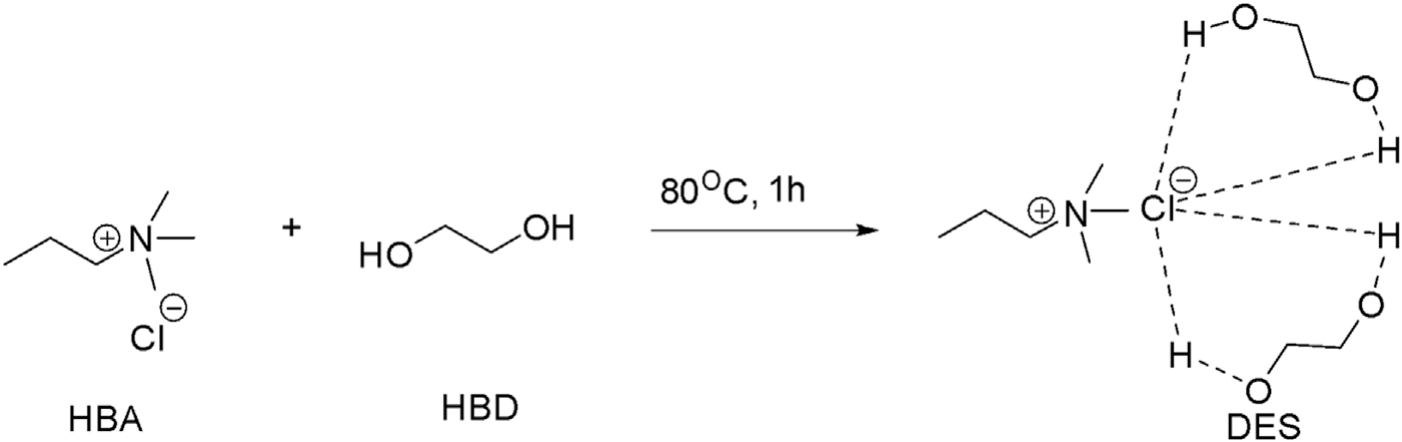
Synthesis of natural deep eutectic solvents (DESs).

Choline chloride and ethylene glycol were taken at a 1:2 stoichiometric ratio, added to a round-bottom flask, and heated for approximately 80°C for 1 h, and the solvent obtained was cooled and used for further synthesis. Chemical changes that occur during the above process are shown in Scheme 1.

### 2.3 Synthesis of NaCoPO_4_ material

**FIGURE 1:**
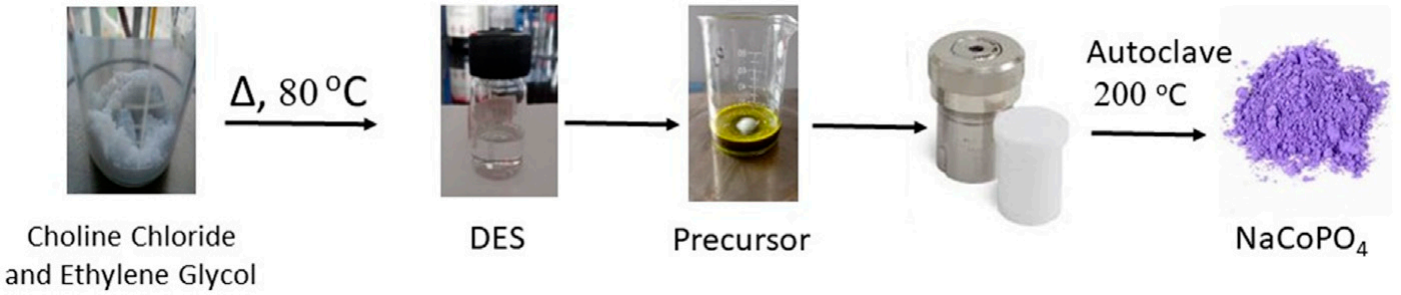
Route for the synthesis of the NaCoPO_4_ material.

The NaCoPO_4_ cathode material was synthesized by an ionothermal method using green solvents such as a deep eutectic solvent. The precursor sources of Na, Co, and phosphate were taken in proportions of 1:2:2. All the precursors were weighed as per the stoichiometric ratios and dissolved in 10 mL of the deep eutectic solvent synthesized in the previous step. The mixture was stirred for approximately 1 h until all the chemicals were thoroughly mixed, poured into a Teflon-lined stainless-steel autoclave (20 mL), and heated for approximately 200°C for 24 h in a programmable muffle furnace, as shown in Figure1. The temperature ramp was maintained at approximately 5°C/min in an air atmosphere. After heating, the autoclave was cooled to room temperature. The solid product obtained was washed with deionized water and then with ethyl alcohol to remove all the unreacted precursors and solvents because all the reactants were soluble in water, so the unreacted precursor and the excess amount of DES after the reaction will be removed using water and ethanol wash, which provides a gray liquid due to the solubility of unreacted precursors. Our title compound is insoluble in water and ethanol, and washing was repeated until the reactants were completely removed. The obtained product was dried at 60°C overnight in a vacuum oven, resulting in a violet solid compound (Shiprath et al., 2021).

### 2.4 Characterization details

The crystal structure was identified using X-ray diffraction using an Ultima IV X-ray diffractometer Cu, ceramic glass, and 2.2 KW. The elemental composition and morphology of the sample were determined by using field emission scanning electron microscopy (FESEM) (JEOL JSM-7100F) and energy-dispersive X-ray (EDX) spectroscopy. Electrochemical studies were performed using a VSP potentiostat–galvanostat instrument, BioLogic, France.

### 2.5 Electrode preparation

The cathode material was prepared using NaCoPO_4_, acetylene black, and polytetrafluoroethylene (PTFE) binder at a ratio of 70:20:10, and the mixture was grounded well in a mortar. The mixture was then transferred to a small-round bottom flask, and a few drops of N-methyl-2- pyrrolidone (NMP) were added and stirred overnight to enhance the conductive and adhesion properties. A Ti-mesh current collector was thoroughly cleaned with distilled water, followed by acetone, dried in vacuum overnight, and weighed before coating it with the cathode slurry. After coating, it was subjected to overnight drying in a vacuum oven for approximately 90°C, and stoichiometry was used during the preparation of an anatase electrode.

## 3 Results and discussion

**FIGURE 2:**
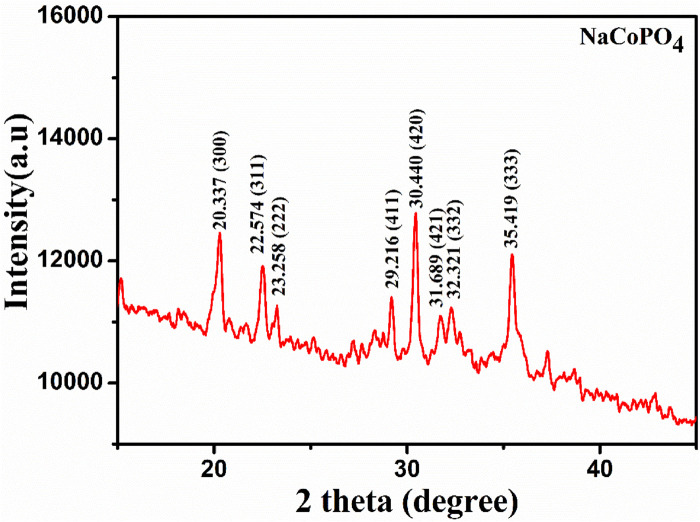
X-ray diffraction pattern of NaCoPO_4_.

The X-ray diffraction (XRD) pattern of the prepared NCP is shown in Figure 2. The diffraction peaks at 2θ = 20.337, 22.574, 23.258, 29.216, 30.440, 31.689, 32.321, and 35.419 correspond to the lattice planes (300), (311), (222), (411), (420), (421), (332), and (333), respectively, and were indexed to the orthorhombic ***β***-NaCoPO_4_. The lattice parameters were determined to be a = 13.08942 Å, b = 13.05091 Å, and c = 15.06989 Å. The cell volume was 2574.4 Å, with no Na^+^ ion (de) intercalation detected prior to the electrochemical studies, which helps in the easy diffusion of sodium ions during charging and discharging mechanisms (Luong et al., 2020; Tripathi et al., 2013). The high-intensity peaks were considered for calculating the average crystalline size, which was calculated using the Debye Scherrer formula to be approximately 25 nm. The crystal structure of ***β***-NaCoPO_4_ is described as a closed framework composed of octahedral CoO_6_ edge-sharing chains and tetrahedral PO_4_ cross-linked chains running parallel to the b-axis.

**FIGURE 3:**
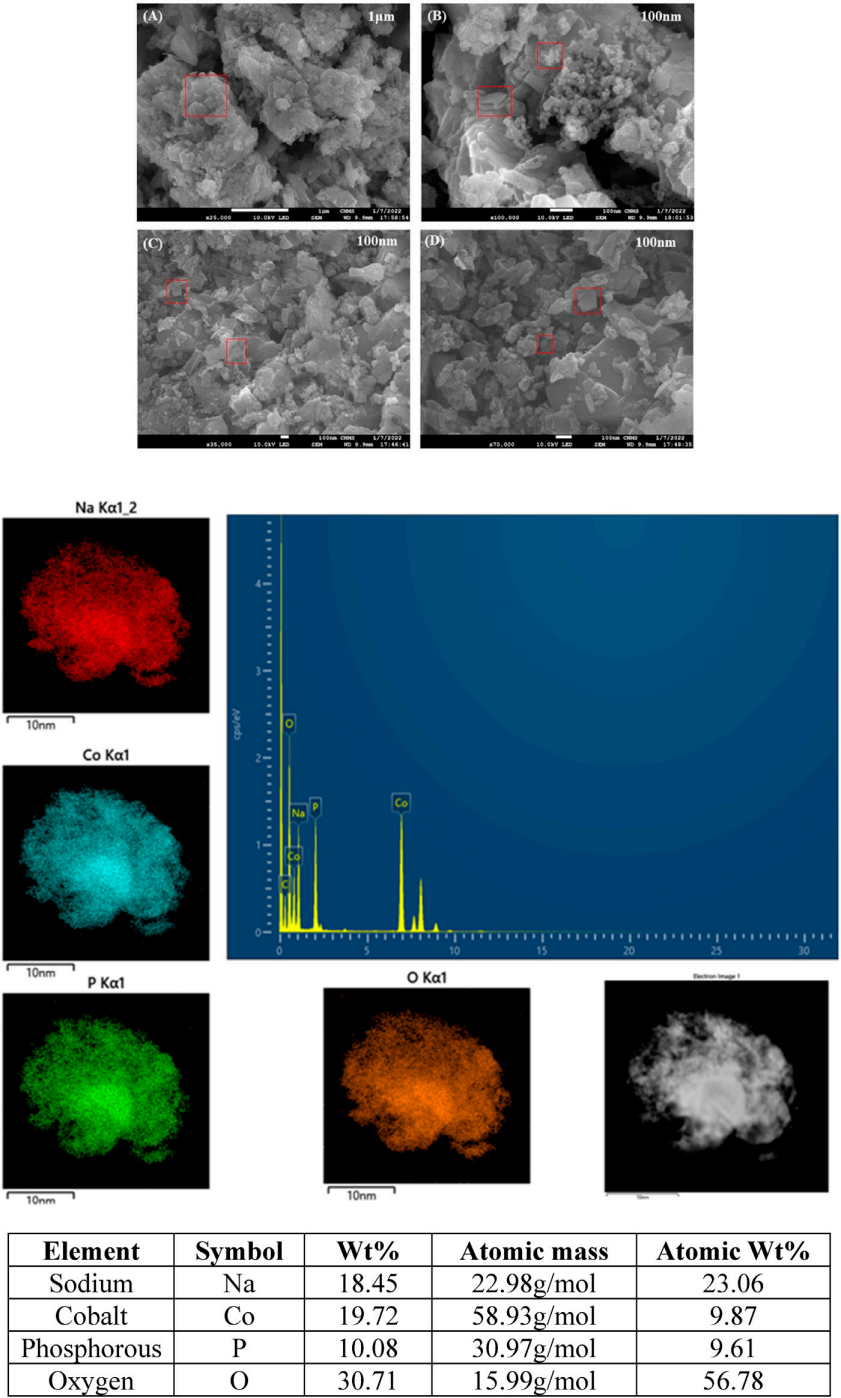
SEM Images **(A–D**) of NaCoPO_4_ with various magnifications with elemental mapping and EDX data representing the percentage of elements.

The surface morphology and structure of the synthesized cathode material (NaCoPO_4_) were obtained using FESEM with different magnifications, and the average particle size calculated from the SEM image was 50 nm, as shown in Figure 3A. The SEM images showed that the material has orthorhombic-shaped particle morphology with uniform distribution and is in good agreement with the powder XRD (PXRD) and elemental mapping images, as shown in Figure 3B, which shows the elemental dispersive x-ray spectroscopy spectrum of NaCoPO_4_ used to determine the elemental composition and surface analysis, which indicates that the uniform distribution of elements (Na, P, Co, and O) throughout the material is interconnected. The percentage composition of elements with less uncertainty <10 was calculated using the formula (Uncertainty % = (Error/Measured) x 100) mentioned in the reference. Therefore, one can understand that the uncertainty can be found most in the case of EDX data. In addition, the little deviation in the atomic percentage of elements can be ascribed to the fact that it is difficult to obtain accurate percentages of elements for these materials containing oxygen. Both the XRD and EDX results show that the NaCoPO_4_ nanomaterial was successfully synthesized via a low-temperature ionothermal method (Shiprath et al., 2020).

**FIGURE 4:**
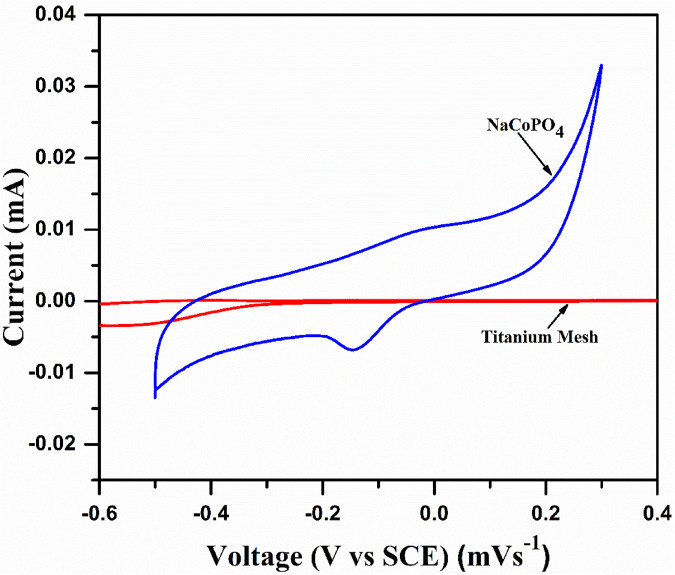
Cyclic voltammogram of titanium mesh as the current collector and NaCoPO_4_ in 2M Na_2_SO_4_ at a scan rate of 0.1 mVs^–1^.

**FIGURE 5:**
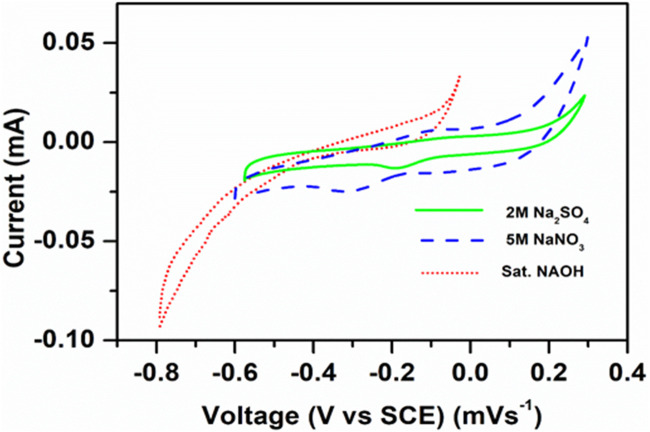
Cyclic voltammograms of NaCoPO4 in different electrolytes such as Na_2_SO_4_, NaNO_3_, and NaOH at 0.1 mVs^–1^.

The electrochemical characterization of the synthesized cathode material was performed with a loading amount of 12 mg/cm^2^, with platinum as the counter electrode and a saturated calomel electrode as the reference electrode with a polypropylene separator in different aqueous sodium electrolytes. A significant peak current enhancement was obtained in 2M Na_2_SO_4_ electrolyte compared to other electrolytes, as shown in Figure 5. Thus, all the other electrochemical investigations (cyclic voltammetry and galvanostatic charge–discharge experiments) were performed using 2M Na_2_SO_4_ electrolyte. Figure 4 shows the CV curve of the current collector (titanium mesh) at 0.15 mVs^−1^ and a broad peak at −0.5 V representing the O_2_ evolution at the current collector and the cyclic voltammogram profile of NaCoPO_4_ recorded in 2 M Na_2_SO_4_. It shows oxidation and reduction peaks at 0.0360 V and at −0.265 V, respectively, vs. the standard calomel electrode with a formal potential of 0.150 V. Both the oxidation and reduction peaks are well within the stable potential window of the Ti-mesh current collector.

The CV studies revealed that the electrochemical redox behavior was effectively observed in the case of the Na_2_SO_4_ solution, as shown in Figure 5.

In the set of redox peaks, a charging and discharging peak was obtained due to the movement of electrons between the cobalt species Co^2+^ and Co^3+^ accompanied by the insertion and de-insertion of the Na^+^ ions in the layered phosphate materials like Na_2_FePO_4_F and NaVOPO_4_ (Good enough J. B, 2013, Tripathi, 2013; Fang et al., 2018) by the following chemical reaction in NaCoPO_4_:

**Charging**: NaCoPO_4,_ → Na_(1-x)_CoPO_4_ + xNa^+^ + xe^-^

**SCHEME 2:**
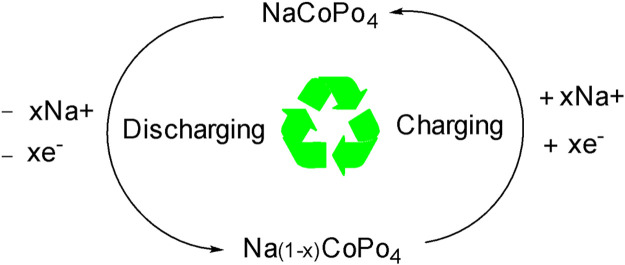
Charging and discharging reactions occurring with the NaCoPO_4_ cathode material.

**Discharging**: Na_(1-x)_CoPO_4_ + xNa^+^ + xe^-^ → NaCoPO_4_

and it is shown schematically as follows in Scheme 2.

The electrochemical behavior of NaCoPO_4_ also varied with different electrolytes, as shown in Figure 5, which clearly indicates that broad peaks were obtained with saturated NaOH due to its high viscosity and low conductivity and the peak shift toward the negative potential side, and it is quite the opposite in the case of 2M Na_2_SO_4_ electrolyte and 5M NaNO_3_ (i.e., positive direction). In general, ideal cathode materials always show redox peaks at the positive side of the potential or at least away from the anodic peak potential. However, the peak currents in 2M Na_2_SO_4_ were moderate compared to 5M NaNO_3_, and the potential was reduced between two redox peaks. It was located on the positive side and the oxidation peak and reduction peak observed at 0.30 V and 0.230 V, respectively, with reference to the standard calomel electrode (SCE), which is significant evidence for the performance of the cathodic material in 2M Na_2_SO_4_ electrolyte, and the other two electrolytes (NaNO_3_ and NaOH) showed quasi-reversible redox behavior, as shown in Figure 5.

**FIGURE 6:**
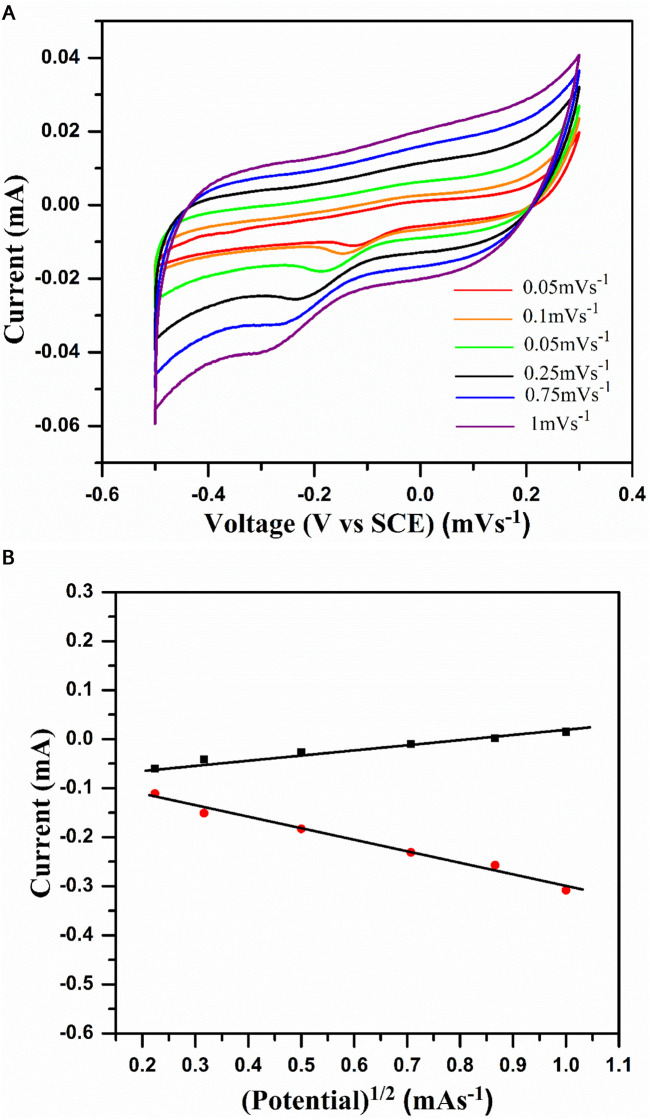
**(A)** Cyclic voltammogram of NaCoPO_4_ in 2M Na_2_SO_4_ solution at different scan rates (0.05, 0.1, 0.25, 0.5, 0.75, and 1). **(B)** Graph representing peak current density vs square root of the scan rate in the 2M Na_2_SO_4_ solution.

To investigate the reversibility of the system, CV studies were carried out by varying the scan rate from 50 μV s^−1^ to 1 mV s^−1^, as shown in Figure 6A. In general, reversible redox systems obey the following conditions: a) the anodic and cathodic peak current magnitude should be the same, i.e., *ipc/ipa* = 1; b) the cathodic and anodic peak current should be a linear function of the square root of the scan rate, i.e., *ipc or ipa* α γ*1/2*; and c) the potential difference with respect to the peak should be 0.059 V.

Due to the inherent properties of the electrodes, they will not obey all the above three rules, and little deviation occurs in their standard behavior. From Figure 6A, the following points can be observed: a) with an enhancement in the scan rate, the peak separation (∆*Ep*) increases with the same trend in the CV profiles; b) peak currents (*i_pa_* and *i_pc_*) exhibit linear dependence with the potential as the scan rate is enhanced; and c) the CV profiles overlapped at the beginning of discharging and charging and are independent of the scan rate. One can understand from the above two points that current is a linear function of the potential of the electrode. Figure 6B shows the plot of cathodic and anodic peak current vs. the square root of the scan rate. Moreover, the linear dependence of peak currents, *i_p_* and *υ^1/2^*, fulfill the third condition (i.e., *i_p_* α *υ^1/2^*) for the redox system, which is reversible at 25°C. However, at the peak current ratio, i.e., *i_pa_/i_pc_* ≠ 1, the reversibility of a redox system is not completely fulfilled. In general, most of the redox systems with resistive behavior will show this trend, which indicates that Na^+^ ion insertion is controlled by the diffusion process in the cathode material.

### 3.1 Identification of the cation

For aqueous electrolyte-based rechargeable sodium-ion electrode materials, it is necessary to detect the kind of ion (sodium or hydrogen) involved in the redox process (insertion and de-insertion), where one should consider the Nernst law that states that redox reactions are always influenced by the formal potential (*E_f_ = E_pa_–E_pc_/2*, where *E_pa_* and *E_pc_* are the anodic and cathodic peak potentials, respectively) of the system depending on the activity of the sodium ion, as represented in the below equation.

*E*_f_ = *E°* + 0.059 log a_Na_^+^.

**FIGURE 7:**
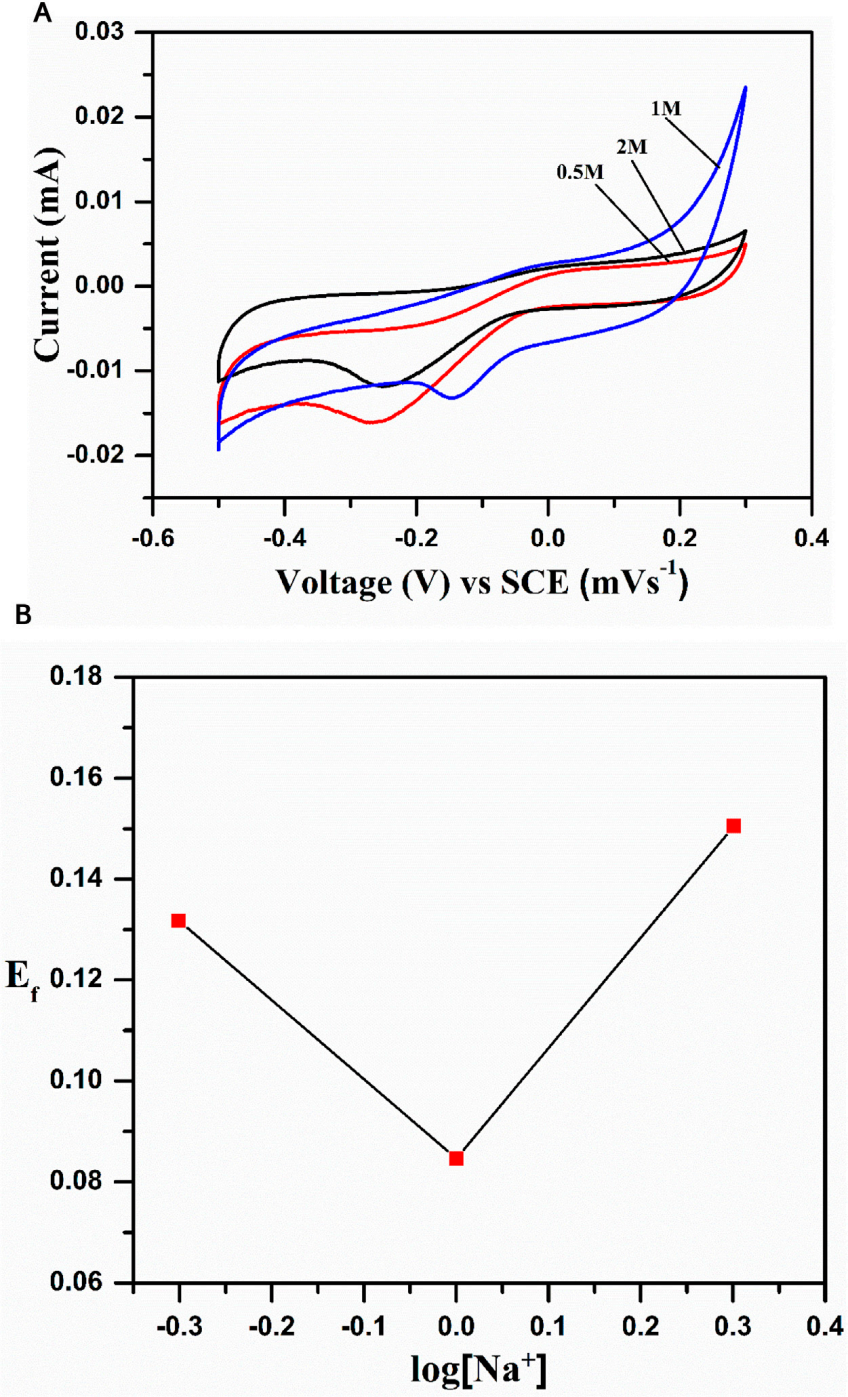
**(A)** Cyclic voltametric curves of NaCoPO_4_ at different concentrations of Na_2_SO_4_ (0.5M, 1M, and 2M solution). **(B)** Graph representing peak current density vs square root of the scan rate in the 2M Na_2_SO_4_ solution.

The above equation shows that the formal potential of the redox system is proportional to the log [Na^+^], which was supported by the CV studies of the system at various concentrations of 0.5 M, 1 M, and 2 M. Figure 7A shows that the oxidation peak potential appears at 0.036 V and reduction peak potential at −0.265 V, with a formal potential of 0.150 V. The formal potential increased with an increase in the concentration of the electrolyte from 0.5 to 1 M, and a prominent change was observed in the CV profiles with a clear positive peak shift and increase in current. However, the trend was changed (peak current is reduced) when we used 2 M electrolyte. To know the nature of the inserted cation, we plotted a graph between formal potential (E_f_) and log [Na^+^], as shown in Figure 7B, which indicates the interference of the H^+^ ion with the Na^+^ ion process at a low concentration. The interference is negligible at a higher concentration (2 M), i.e., anodic and cathodic peak currents reduced, as shown in Figure 7B.

**FIGURE 8:**
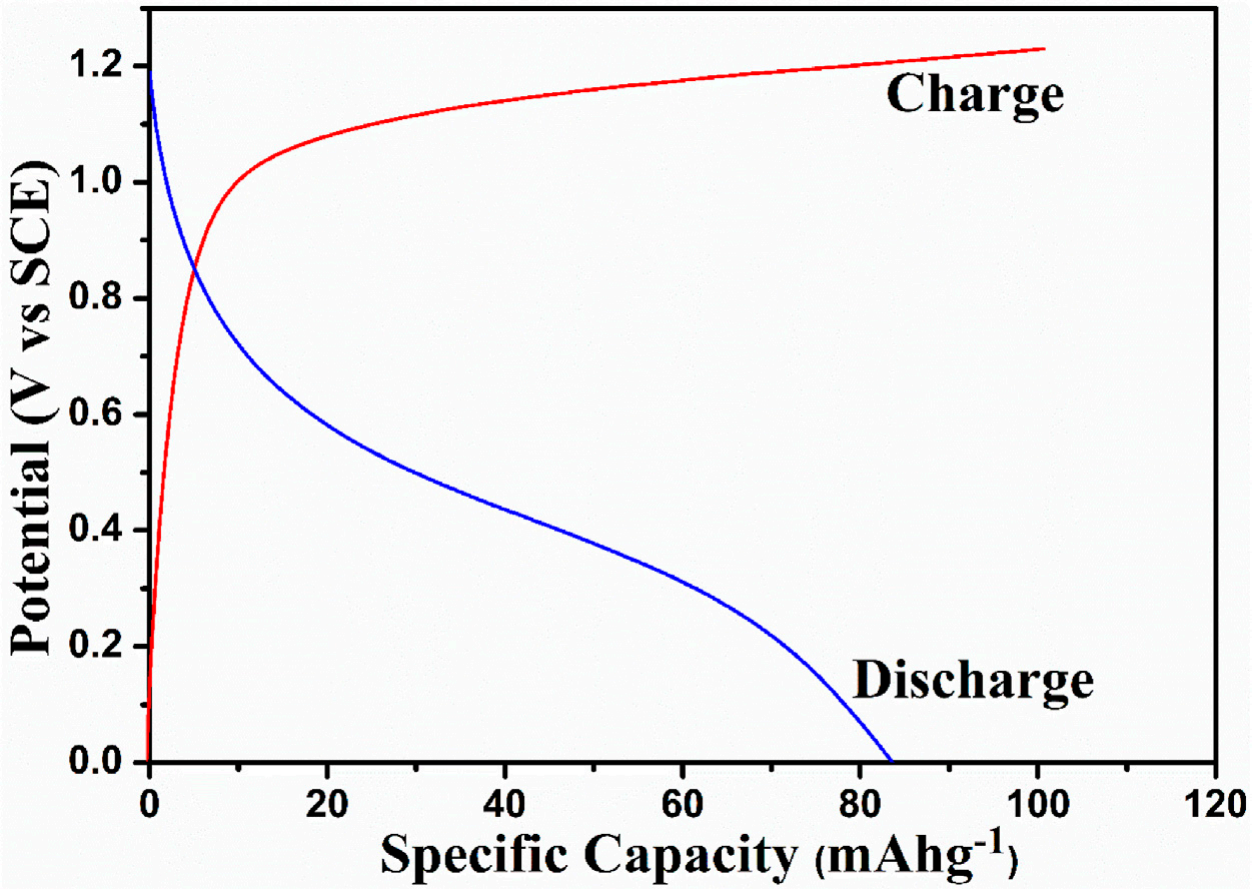
Charge–discharge curves of NaCoPO_4_/TiO_2_ in the 2M Na_2_SO_4_ solution.

The charge–discharge studies were performed in the voltage range 0 V–1.5 V with the TiO_2_/2MNa_2_SO_4_/NaCoPO_4_ cell system, as shown in Figure 8. A discharge capacity of 85 mAh^−1^ was obtained with an applied current of 0.2 mA. The material exhibited better discharge capacity than the other materials, as shown in Table 1.

**TABLE 1:** Comparison of the discharge capacity of our material with reported compounds.

S. no.	Material	Synthetic method	Discharge capacity	Reference
1	Na_3_Fe_3_(PO_4_)_4_	Sol–gel	80 mAh g^−1^	Qiu et al. (2021)
2	Fe_3_O_4_@rGO composite	Solvo-thermal	80 mAh g^−1^	Lu et al. (2015)
3	GO-FeSe_2_	Hydrothermal	60 mAh g^−1^	Li et al. (2022)
4	NaCr[Fe (CN)_6_]	Co-precipitation method	64 mAh g^−1^	baster et al. (2019)
5	Na_2.85_K_0.15_V_2_(PO_4_)_3_	Solvo-thermal	52 mAh g^−1^	Shen et al. (2020)
6	NaCoPO_4_	DES-assisted ionothermal	85 mAh g^−1^	Current work

Moreover, a slight inflection is observed at 0.5 V during the discharge process, which is caused by the twinning effect with the insertion of an orthorhomboidal structure. This is not the case during the charging process, but a constant decrease in the potential with a discharge cutoff up to 0 V is observed.

**FIGURE 9:**
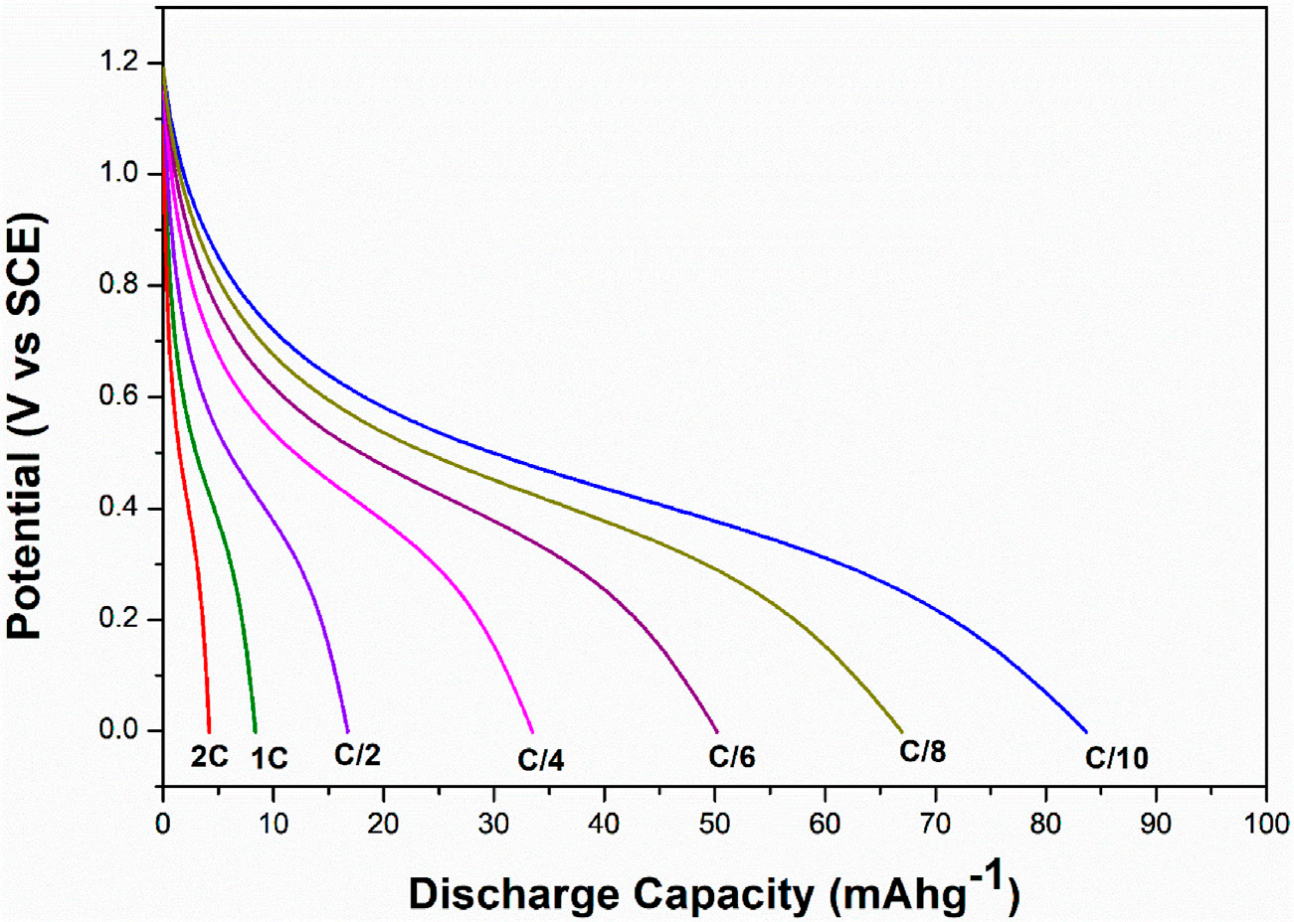
Discharge curves of NaCoPO_4_/TiO_2_ at different C-rates in the 2M aqueous Na_2_SO_4_ electrolyte.

**FIGURE 10:**
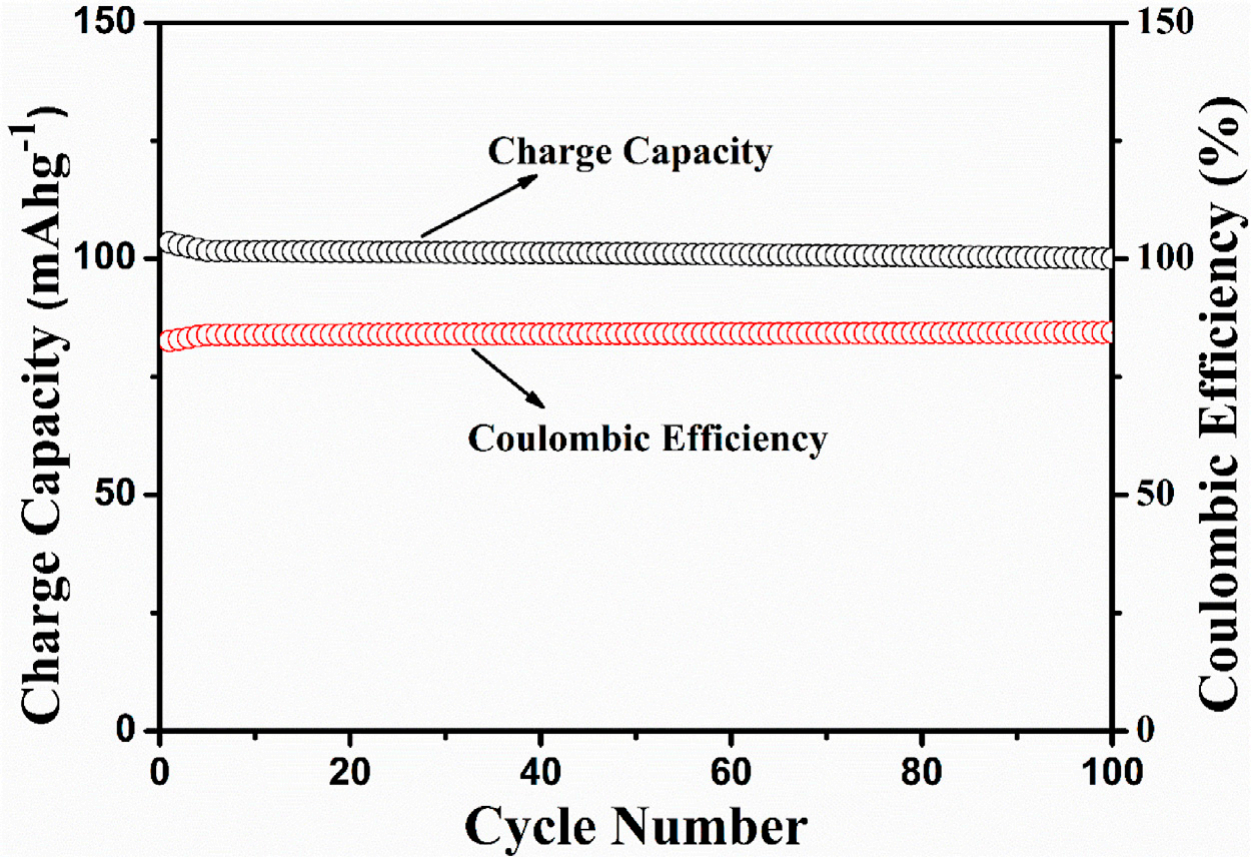
Charge capacity vs no. of cycles and coulombic efficiency vs the no. of cycles.

**FIGURE 11:**
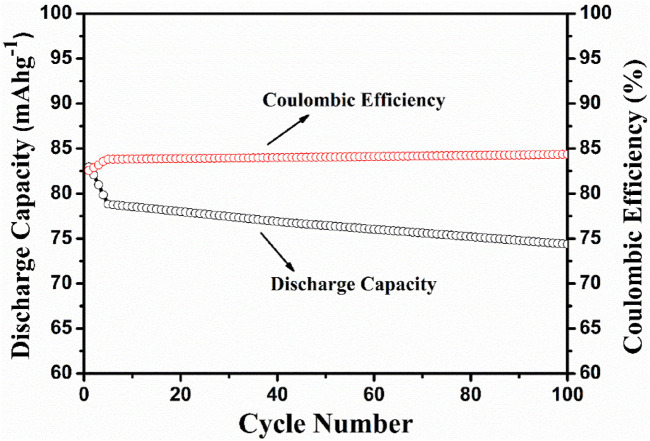
Discharge capacity vs no. of cycles at the C/10 rate and coulombic efficiency vs the no. of cycles.

The electrochemical cell anatase TiO_2_/2MNa_2_SO_4_/NaCoPO_4_ is performed with charge–discharge cycle studies at various C rates, as shown in Figures 9, 10. The percentage of the theoretical capacity is retained at a C/2 rate. The coulombic efficiency and the capacity are measured for about 100 cycles, as shown in Figure 10, and it indicates that the efficiency of the cell increased during the first few cycles, and the discharge capacity decreased by 10% and is gradually retained in further cycles, as shown in Figure 11.

## 4 Conclusion

NaCoPO_4_ and anatase-based aqueous rechargeable sodium-ion batteries are cost-effective and easily compete with the highly successful lead–acid aqueous storage battery with proper optimization. The synthesized cathode material was shown to have a moderate and constant coulombic efficiency with a discharge capacity of approximately 75 mAh^−1^ up to 100 cycles, with simple conducting additives such as acetylene black. Moreover, it shows greater reversibility and motivates us to improve the discharge capacities with proper optimization using various carbonaceous conducting materials such as reduced graphene oxide and Multiwalled Carbon Nanotubes (MWCNTs) as support to this material. Thus, the prepared materials are suitable candidates for rechargeable Na-ion batteries.

## Data Availability

The original contributions presented in the study are publicly available. This data can be found here: https://github.com/janaorm2011/1440639-XRD-Files. Further inquiries can be directed to the corresponding author.
